# Co‐designing and implementing the GreenConnect Dementia Respite Program to improve quality of life of the people living with dementia through nature‐based activities

**DOI:** 10.1002/alz70858_097356

**Published:** 2025-12-24

**Authors:** Shahinoor Akter, Sean MacDermott, Irene Darmadi Blackberry, Dan Douglass, Gerard José, Anthony Couroupis, Jim Boyer, Tshepo Rasekaba

**Affiliations:** ^1^ John Richards Centre for Rural Ageing Research, La Trobe Rural Health School, La Trobe University, Albury‐Wodonga, VIC, Australia; ^2^ Care Economy Research Institute, La Trobe University, Albury‐Wodonga, VIC, Australia; ^3^ La Trobe University, Wodonga, VIC, Australia; ^4^ Heathcote Health, Heathcote, VIC, Australia; ^5^ Rural Care Australia, Mildura, VIC, Australia; ^6^ Princes Court Ltd, Mildura, VIC, Australia; ^7^ Heathcote Dementia Alliance, Heathcote, VIC, Australia

## Abstract

**Background:**

With the increased demand for respite services, innovative solutions are critical to effectively address the complex needs of individuals living with dementia and their carers. Dementia‐friendly respite involving green environment (i.e., Green Care) can improve the quality of life (QoL) of people living with dementia and reduce carer burden. This abstract presents the implementation of a dementia‐specific respite program, the GreenConnect Dementia Respite Program, in rural Victoria, Australia. Drawing upon Green Care principles, this Program offers nature‐based respite activities, experiences and accommodation away from residential aged care (RAC) settings aimed at improving QoL for the target population.

**Methods:**

In the co‐design phase (November 2023 and January 2024), we conducted a consultation workshop and two focus group discussions (FGDs) to facilitate participations of key stakeholders in the development of effective implementation strategies, identifying potential partners to provide accommodation and activities related to the project and to develop an evaluation plan. Participants (*N* = 35) including people living with dementia, carers, service providers, research academics and advocacy groups participated in the discussions. Following a thematic analysis of the data underpinning socio‐ecological framework, the Program's model of care and implementation strategies were developed. The Program commenced in April 2024 and will continue until June 2026 with an aim to engage up to 250 people living with dementia and carers in the Program. Trained and experienced Care Coordinators are implementing nature‐based respite activities outside RAC. Using a RE‐AIM framework, the program is also undergoing mixed‐methods evaluation including surveys (DEMQOL, Zarit Burden interviews and satisfaction surveys), interviews with participants and FGDs with staff.

**Results:**

The implementation of the Program is ongoing and at the time of abstract development, a total of 50 people living with dementia and carers were assessed and participated in 16 activities. Progress and learning of our implementation study to date will be shared at the conference upon acceptance.

**Conclusion:**

This is the first nature‐based dementia respite program in rural Australia aimed at addressing both evidence and service gaps in dementia care. Insights gained from the implementing this Program may facilitate the integration of nature‐based approaches into respite care services.

